# The complete chloroplast genome sequence of *Blastus pauciflorus* (Melastomataceae)

**DOI:** 10.1080/23802359.2019.1687035

**Published:** 2019-11-06

**Authors:** Zhen Ying Wen, Si Jin Zeng, Wenchao Han, Bin Chen, Dong Hui Peng

**Affiliations:** a Fujian Agriculture and Forestry University, Fuzhou, China;; b South China Agricultural University, Guangzhou, China;; c The National Orchid Conservation Center of China, The Orchid Conservation and Research Center of Shenzhen, Shenzhen, China

**Keywords:** *Blastus pauciflorus*, Melastomataceae, chloroplast genome, illumina sequencing, phylogenetic analysis

## Abstract

*Blastus pauciflorus*, a shrub endemic to Hong Kong and Guangdong, south China, growing on low-altitude hillsides, under the forest. The species is controversial in classification. Herein, we report the complete chloroplast genome sequence assembled from Illumina pair-end sequencing data, with aims to resolve its relationship with the related species. The complete chloroplast genome was 155,983 bp in length, includes two inverted repeat regions (IRs) of 26,716 bp each, which were separated by a large single copy region (LSC) 86,101 bp and a small single copy region (SSC) 16,450 bp. The chloroplast genome contained 129 genes, including 82 protein-coding genes, 2 pseudogenes, 37 tRNA genes and 8 rRNA genes. The overall GC content in the chloroplast genome of *B. pauciflorus* was 37.0%. Phylogenetic analysis showed that *B. pauciflorus* is closed to *B. cochinchinensis*.

*Blastus pauciflorus* (Benth.) Guillaum, belonging to the family Melastomataceae, is a shrub endemic to Guangdong, China (Chen 1984), only growing on the low altitude hillside forest. It is characterized by densely yellow glandular on abaxial leaf surface; inflorescences terminal, panicled cymose, densely puberulous and remotely glandular; anthers ca. 3 mm long, base obtuse, slightly divided; sepals short triangular, <1 mm long. *Blastus pauciflorus* is poorly understood, resulting in great difficulties in identifying. Chen and Renner (2007) incorporated five species and three variations (*B. longiflorus* Hand.-Mazz., *B. longiflorus* var. *apricus* (Hand.-Mazz.) Y. L. Zheng & N. H. Xia, *B. cavaleriei* H. Lév., *B. cavaleriei* var. *tomentosus* (H. L. Li) C. Chen, B. *dunnianus* H. Lév., *B. dunnianus* var. *glandulosetosus* C. Chen, B. *ernae* Hand.-Mazz., and *B. squamosus* C. Chen) under *B. pauciflorus* into the genus *Blastus*, because of the lack of molecular data, the validity of some of these merges are debatable. To better understand the evolution history and genetic information of *Blastus*, the complete chloroplast genome sequence was characterized.

The fresh leaf sample was acquired from Hong Kong, China, where was the type locality. And the voucher specimen (specimen code WZY073) was deposited at the Herbarium of College of Forestry, Fujian Agriculture and Forestry University (FJFC). The total genomic DNA was extracted from fresh leaves using Plant Tissues Genome DNA Extraction Kit (TianGen DP305, Beijing, China), and sequenced using the Illumina pair-end technology. The clean reads were firstly aligned to *Blastus cochinchinensis* (GenBank accession MK814186) and then assembled using the software GetOrganelle (Jin et al. 2018). The assembled chloroplast genome was annotated using DOGMA, and the annotation was corrected using Geneious R10 (Biomatters Ltd., Auckland, New Zealand) (Kearse et al. 2012). The annotation result was drawn using OGDRAW (Lohse et al. 2013). The accurate new annotated complete chloroplast genome of *B. pauciflorus* was submitted to GenBank (accession number MN517839). The complete chloroplast genome of *B. pauciflorus* was a circular molecule of 155,983 bp, containing of a large single-copy (LSC) region of 86,101 bp and a small single-copy (SSC) region of 16,450 bp, separated by a pair of inverted repeat region of 26,716 bp each. The genome contained 129 genes, including 82 protein-coding genes, 2 pseudogenes, 37 transfer RNA (tRNA) genes, and 8 ribosomal RNA (rRNA) genes. The overall GC content was 37.0%.

For phylogenetic tree reconstruction, the chloroplast genome sequences of *B. pauciflorus* was aligned with 20 other complete chloroplast genomes of Melastomataceae (Reginato et al. 2016; Ng et al. 2017; Zhou et al. 2018; Wen et al. 2019; Zhang et al. 2019), and *Eucalyptus globulus* (GenBank accession AY780259) as an outgroup. The sequences were aligned using HomBlocks pipeline (Bi et al. 2018). A maximum-likelihood phylogenetic tree (Figure 1) was constructed with RAxML-HPC2 on XSEDE version 8.2.10 (Stamatakis 2014), the branch support was computed with 1000 bootstrap replicates. The phylogenetic analysis showed that *B. pauciflorus* is closest to *B. cochinchinensis* in Melastomataceae.

**Figure 1.**
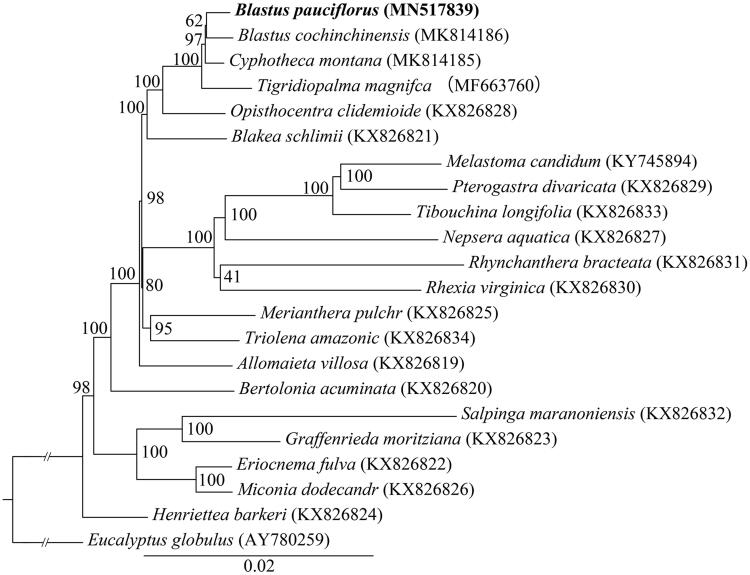
Maximum-likelihood tree from the analysis of 20 complete chloroplast genome of Melastomataceae, with *Eucalyptus globulus* as an outgroup. The bootstrap support values shown next to the nodes were based on 1000 replicates.
